# The confounding effect of cryptic relatedness for environmental risks of systolic blood pressure on cohort studies

**DOI:** 10.1002/mgg3.4

**Published:** 2013-04-03

**Authors:** Kyoko Shibata, Atsushi Hozawa, Gen Tamiya, Masao Ueki, Tomohiro Nakamura, Hiroto Narimatsu, Isao Kubota, Yoshiyuki Ueno, Takeo Kato, Hidetoshi Yamashita, Akira Fukao, Takamasa Kayama, Yamagata University Genomic Cohort Consortium

**Affiliations:** 1 Advanced Molecular Epidemiology Research Institute (AMERI), Cohort Management Unit, Yamagata UniversityYamagata, Japan; 2 Division of Personalized Prevention and Epidemiology, Department of Preventive Medicine and Epidemiology, Tohoku Medical Megabank Organization, Tohoku UniversitySendai, Japan; 3 AMERI, Genomic Information Analysis Unit, Yamagata UniversityYamagata, Japan; 4 AMERI, Cohort Management Unit, Yamagata UniversityYamagata, Japan; 5 AMERI, Respiratory and Cardiovascular Diseases Research Center, Yamagata UniversityYamagata, Japan; 6 Department of Cardiology, Pulmonology, and Nephrology, Yamagata UniversityYamagata, Japan; 7 Department of Gastroenterology, Yamagata UniversityYamagata, Japan; 8 AMERI, Metabolic and Degenerative Diseases Research Center, Yamagata UniversityYamagata, Japan; 9 Department of Neurology, Hematology, Metabolism, Endocrinology and Diabetology, Yamagata UniversityYamagata, Japan; 10 Department of Ophthalmology, Yamagata UniversityYamagata, Japan; 11 Graduate School of Medicine, Department of Neurosurgery, Yamagata UniversityYamagata, Japan

**Keywords:** confounding effect, cryptic relatedness, systolic blood pressure

## Abstract

The impact of cryptic relatedness (CR) on genomic association studies is well studied and known to inflate false-positive rates as reported by several groups. In contrast, conventional epidemiological studies for environmental risks, the confounding effect of CR is still uninvestigated. In this study, we investigated the confounding effect of unadjusted CR among a rural cohort in the relationship between environmental risk factors (body mass index, smoking status, alcohol consumption) and systolic blood pressure. We applied the methods of population-based whole-genome association studies for the analysis of the genome-wide single nucleotide polymorphism data in 1622 subjects, and detected 20.2% CR in this cohort population. In the case of the sample size, approximately 1000, the ratio of CR to the population was 20.2%, the population prevalence 25%, the prevalence in the CR 26%, heritability for liability 14.3% and prevalence in the subpopulation without CR 26%, the difference of estimated regression coefficient between samples with and without CR was not significant (*P*-value = 0.55). On the other hand, in another case with approximately >20% heritability for liability, we showed that confounding due to CR biased the estimation of exposure effects.

## Introduction

Cryptic relatedness (CR) is well known as a confounding factor in genome wide association study (GWAS) (Yu et al. 2006; Kang et al. 2008; Price et al. 2010), which inflates the false-positive rate. Voight and Pritchard (2005) developed a formal model of CR and studied its impact on genomic case–control association studies. They showed that the degree of confounding due to CR would usually be negligible. However, in contrast, they also reported on studies with sampling biases toward collecting relatives may indeed suffer from excessive rates of false positives. Typically, epidemiological designs in which individuals are ascertained nonrandomly from a closed population, the effect of the influence of close relatives might not be negligible. It is therefore important to correct or account for the confounding effect of CR in epidemiological cohort studies that have collected data from a limited or small-sized sample. However, the knowledge of the confounding effect of CR in epidemiological association studies is still unknown. Here, we examined the confounding effect of CR in the relationship between systolic blood pressure (SBP) and several environmental risk factors (body mass index [BMI], smoking status [daily smoker vs. nonsmoking], and alcohol consumption [drinking vs. abstention]).

It is also well known that being overweight and obese increases the risk of high blood pressure (Kannel 1967; World Health Organization 2000). However, interpreting the blood pressure–BMI relationship is further complicated by data from other studies, in which there appears to be no correlation between these variables (Roche and Siervogel 1991; Spiegelman et al. 1992; Bunker et al. 1995; Gallagher et al. 1996). In this article, we examined whether the confounding effect of CR might involve in the relationship between blood pressure and BMI. Additional goals of the study were to assess the confounding effect of CR in any potential relationship between blood pressure and the risk of other environmental factors; for example, smoking and alcohol consumption. There are several studies that examined the relationship between alcohol consumption or smoking and blood pressure in a Japanese population (Kiyohara et al. 1995; Minami et al. 2002; Ohmori et al. 2002). Ohira et al. (2009) looked into the effect of habitual alcohol intake on ambulatory blood pressure among Japanese men, which was associated with increased BP in the morning. Minami et al.(1999) studied the effects of smoking cessation on blood pressure in habitual smokers. However, there were no studies which examined the confounding effect of CR in the relationship between blood pressure and the risk of environmental factors. We aim to address the question of whether CR is likely to be a serious issue for inferring epidemiological relationship between these factors using the cohort study of Takahata residents. First, using the techniques to detect and correct for unrecognized population structure in GWAS, we examined how CR was presented in the sampling. Next, we tested the assumption of parallel regressions to examine whether its confounding effect as a covariate affected on environmental risk factors in difference setting (sample size, ratio of CR to the population, prevalence in CR, the population prevalence). Then, we applied multiple regression analysis to these data with and without CR in order to examine the differences obtained in estimating the regression coefficient.

## Methods

### Analysis of real data

We used the genome-wide 657,366 single nucleotide polymorphism (SNP) data and SBP as a phenotype in the cohort study of Takahata. We selected BMI, variables for smoking status (1: nonsmoking vs. 2: daily smoker), and alcohol consumption (1: abstention vs. 2: drinking) as environmental risk factors, and gender (1: male; 2: female) and age as covariates. Weight, height, and SBP were measured and standardized in the Takahata cohort design. We examined the relationship between individuals genetic background by the PLINK (Purcell et al. 2007) and the EIGENSTRAT methods (Price et al. 2006). We detected relationships between subjects using identity by descent (IBD) probability as a measurement.

### Multiple regression analyses

We sampled our cohort population in a difference setting; sample size (approximately 1000, 400, or 500), the ratio of CR to the population (approximately 20%, 40%, or 50%), the prevalence in the subpopulation without CR (26%, 50%, or 76%), the prevalence in CR (26% fixed), the population prevalence (25%, 40%, or 50%), and heritability for liability (approximately 14%, 22%, or 32%) in our cohort population. Here, we estimated heritability for liability using the formula {(*x*_*p*_ − *x*_*q*_)/*a*_*p*_}/*ρ*, where *ρ* denotes the expected proportion of alleles shared IBD (i.e., *ρ* = 2^−*R*^, where *R* denotes the degree of relationship), *x*_*p*_ denotes the difference between mean value in the subpopulation without CR and threshold, *x*_*q*_ denotes the difference between mean value in the subpopulation without CR and mean value in CR, and *a*_*p*_ denotes the difference between population mean and mean value in the group of affected individuals (Yasuda 2007). In our context, *ρ* can be defined as the sum of expected proportion of allele shared IBD for all of the degree of relationship in our detected CR (i.e., *R* = 0, 1, 2). First, we tested the assumption of parallel regressions to examine whether the confounding effect of CR as a covariate affected on environmental risk factors; that is, the following test was performed,


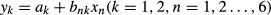


Null hypothesis: *b*_*n*1_ = *b*_*n*2_, alternative hypothesis: *b*_*n*1_ ≠ *b*_*n*2_,

where *k* is the number of population (i.e., population with and without CR), and *n* is the number of independent variables and covariates. Then, we applied multiple regression analysis to these data with and without CR in order to examine the differences obtained in estimating the regression coefficient.

### Cohort description

We performed an analysis of the data collected within a closed, small prospective study, concerned with various risk factors for common diseases.

#### Subject recruitment

The Takahata cohort was established for a baseline survey in a small rural town, Takahata in Yamagata Prefecture from 2006 to 2008. The total population size has been constant, approximately 25,000 throughout this period. The Takahata cohort has become part of our large genomic cohort initiative, the Yamagata cohort, which is now ongoing in the urban prefectural capital, Yamagata City, having approximately 250,000 residents. We used genomic DNAs from 1622 individuals who completed the questionnaire for environmental exposures and informed consent for our modern prospective genomic cohort study. This cohort study was performed under the approval by the Committee on Ethics at Yamagata University and all other institutions involved.

#### Genotyping

Using genomic DNAs from the Takahata population, we carried out genotyping for 657,366 SNPs using the Infinium Assay with Human660W-Quad BeadChip (Illumina, San Diego, CA) according to the standard procedure provided by Illumina.

## Results

**Figure 1:**
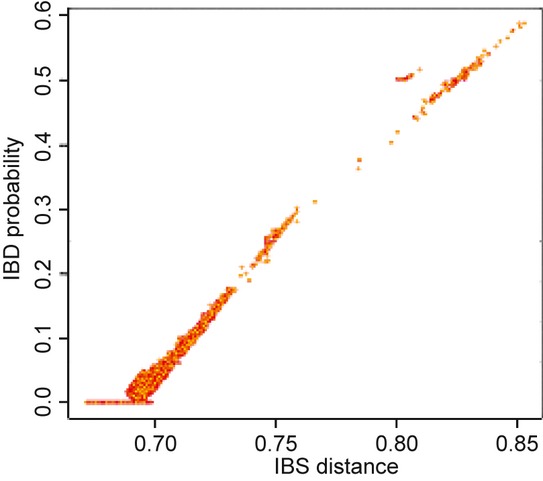
Plot of the relationship between total 1622 subjects with an identity by descent (IBD) probability with regard to an identity by state (IBS) distance; *y*-axis and *x*-axis describe IBD probability and IBS distance, respectively.

**Figure 2:**
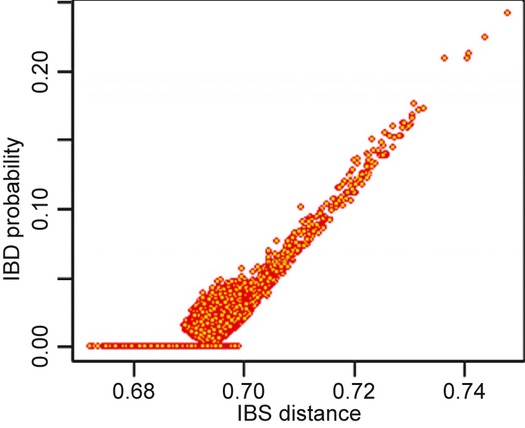
Plot of the relationship between 1291 subjects with an identity by descent (IBD) probability with regard to an identity by state (IBS) distance after which is removed a cryptic relatedness of 326 subjects with an IBD probability >1/4 (i.e., monozygotic twins, dizygotic twins, full-sibs, parent-offspring, half-siblings, grandparent, grandchild, aunt/uncle, and niece/nephew); *y*-axis and *x*-axis describe IBD probability and IBS distance, respectively.


Figure 1 shows the relationship between total 1622 subjects with an IBD probability with regard to an identity by distance. First step, we removed individuals for low genotyping (*P* ≤ 0.05) from total 1622 sample using the PLINK method. By the PLINK and the EIGENSTRAT methods, we detected a relationship between subjects with an IBD probability >1/4 as a CR of 326 subjects (i.e., monozygotic twins, dizygotic twins, full-sibs, parent-offspring, half-siblings, grandparent, grandchild, aunt/uncle, and niece/nephew) in the sample of 1617 subjects. Figure 2 shows the relationship between 1291 subjects with an IBD probability with regard to an identity by distance after removed a CR of 326 subjects. Next, we removed subjects medically treated for blood pressure from samples with and without CR, respectively. Using the sample with and without CR, the sample sizes were 1039 and 829 individuals, respectively. We analyzed the data as described above. In this case, the heritability for liability was 14.3%. In the multiple regression analysis, the regression model found from the sampling data with CR was as follows:

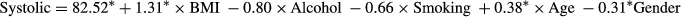
(1)

where “Systolic” denotes systolic blood pressure and * indicates significant, *P*-values <0.05. Note that adjusted *R*-squared for equation (1) was 0.14.

The regression model found from the sampling data without CR was as follows:


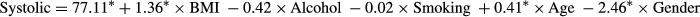
(2)

Note that adjusted *R*-squared for equation (2) was 0.15.

Compared with equations (1) and (2), the results showed that the presence of CR is apparently not affected by estimating regression coefficient in regression modeling (Table 1). We tested the assumption of parallel regressions for equations (1) and (2). The difference between equations (1) and (2) was not significant (*F*-value = 0.35, *P*-value = 0.55).

**Table 1:** Results of the regression coefficients between systolic blood pressure and environmental risk factors in the sample with and without cryptic relatedness (CR): sample size 1039, ratio of CR to the population 20.2%, population prevalence 25%, heritability for liability 14.3%, prevalence in the subpopulation without CR 26%, prevalence in CR 26%

	Estimated	Standard error	*t*-value	Pr(>|*t*|)
Intercept				
Sample with CR^1^	82.52	5.15	16.03	<2e^−16^
Sample without CR^2^	77.11	5.49	14.06	<2e^−16^
BMI				
Sample with CR	1.31	0.14	9.30	<2e^−16^
Sample without CR	1.36	0.16	8.34	3.23e^−16^
Alcohol consumption				
Sample with CR	−0.80	1.51	−0.53	0.60
Sample without CR	−0.42	1.61	−0.26	0.79
Smoking status				
Sample with CR	−0.66	0.71	−0.93	0.35
Sample without CR	−0.02	1.05	−0.02	0.98
Age				
Sample with CR	0.38	0.07	5.43	6.86e^−08^
Sample without CR	0.41	0.05	7.99	4.62e^−15^
Gender				
Sample with CR	−3.09	1.04	−2.97	0.003
Sample without CR	−2.46	1.01	−2.44	0.01

1 Size of sample with CR was 1039 subjects. From the sampling data with CR, equation (1) in Results were found. Adjusted *R*-squared = 0.14.

2 Size of sample without CR was 829 subjects. From the sampling data without CR, equation (2) in Results were found. Adjusted *R*-squared = 0.15.

Furthermore, as described in Methods, we analyzed under the following difference setting, conditional on the fixed prevalence in CR of 26%. The regression model found from the sampling data in the sample size of 400, the ratio of CR to the population 52.5%, prevalence in the population 40%, prevalence in the subpopulation without CR 55%, prevalence in CR 26%, the heritability for liability 24.2% was as follows:


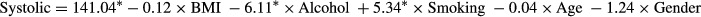
(3)

Note that adjusted *R*-squared for equation (3) was 0.25. In contrast, the regression model found from the sampling without CR (sample size 190) was as follows:


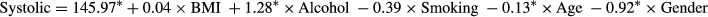
(4)

Note that adjusted *R*-squared for equation (4) was 0.16. Compared with equations (3) and (4), the results showed that the presence of CR is apparently affected by estimating regression coefficient in regression modeling (Table 2). By testing the assumption of parallel regressions for equations (3) and (4), the difference between equations (3) and (4) was significant (*F*-value = 41.83, *P*-value = 2.103e^−10^).

**Table 2:** Results of the regression coefficients between systolic blood pressure and environmental risk factors in the sample with and without cryptic relatedness (CR): sample size 400, ratio of CR to the population 52.5%, population prevalence 40%, heritability for liability 24.2%, prevalence in the subpopulation without CR 55%, prevalence in CR 26%

	Estimated	Standard error	*t*-value	Pr(>|*t*|)
Intercept				
Sample with CR^1^	141.04	5.52	25.56	<2e^−16^
Sample without CR^2^	145.97	1.67	87.40	<2e^−16^
BMI				
Sample with CR	−0.12	0.15	−0.76	0.45
Sample without CR	0.04	0.05	0.79	0.43
Alcohol consumption				
Sample with CR	−6.11	1.81	−3.37	0.0008
Sample without CR	1.28	0.56	2.30	0.022
Smoking status				
Sample with CR	5.34	0.71	7.57	2.69e^−13^
Sample without CR	−0.39	0.25	−1.59	0.11
Age				
Sample with CR	−0.040	0.082	−0.48	0.63
Sample without CR	−0.13	0.02	−5.47	1.44e^−07^
Gender				
Sample with CR	−1.24	1.13	−1.10	0.27
Sample without CR	−0.92	0.32	−2.86	0.004

1 Size of sample with CR was 400 subjects. From the sampling data with CR, equation (3) in Results were found. Adjusted *R*-squared = 0.25.

2 Size of sample without CR was 190 subjects. From the sampling data without CR, equation (4) in Results were found. Adjusted *R*-squared = 0.16.

The regression model found based on the sampling data in the sample size of 400, the ratio of CR to the population 52.5%, prevalence in the population 50%, prevalence in the subpopulation without CR 76.3%, prevalence in CR 26%, the heritability for liability 32.4% was as follows:


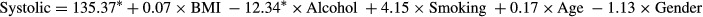
(5)

Note that adjusted *R*-squared for equation (5) was 0.24. In contrast, the regression model found from the sampling without CR (sample size 190) was as follows:


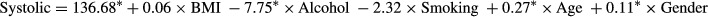
(6)

Note that adjusted *R*-squared for equation (6) was 0.42. Compared with equations (5) and (6), the results showed that the presence of CR is apparently affected by estimating regression coefficient in regression modeling (Table 3). By testing the assumption of parallel regressions for equations (5) and (6), the difference between equations (5) and (6) was significant (*F*-value = 39.74, *P*-value = 5.720e^−10^).

**Table 3:** Results of the regression coefficients between systolic blood pressure and environmental risk factors in the sample with and without cryptic relatedness (CR): sample size 400, ratio of CR to the population 52.5%, population prevalence 50%, heritability for liability 14.3%, prevalence in the subpopulation without CR 76.3%, prevalence in CR 26%

	Estimated	Standard error	*t*-value	Pr(>|*t*|)
Intercept				
Sample with CR^1^	135.37	5.77	23.47	<2e^−16^
Sample without CR^2^	136.68	2.09	65.34	<2e^−16^
BMI				
Sample with CR	0.07	0.16	0.46	0.64
Sample without CR	0.06	0.05	1.11	0.27
Alcohol consumption				
Sample with CR	−12.34	2.00	−6.16	1.77e^−09^
Sample without CR	−7.75	0.81	−9.54	<2e^−16^
Smoking status				
Sample with CR	4.15	0.74	5.62	3.60e^−08^
Sample without CR	−2.32	0.32	−7.23	1.24e^−11^
Age				
Sample with CR	0.17	0.09	1.89	0.06
Sample without CR	0.27	0.03	7.84	3.56e^−13^
Gender				
Sample with CR	−1.13	1.18	−0.95	0.34
Sample without CR	0.11	0.41	0.27	0.79

1 Size of sample with CR was 400 subjects. From the sampling data with CR, equation (5) in Results were found. Adjusted *R*-squared = 0.24.

2 Size of sample without CR was 190 subjects. From the sampling data without CR, equation (6) in Results were found. Adjusted *R*-squared = 0.42.

The regression model found from the sampling data in the sample size of 500, the ratio of CR to the population 42%, prevalence in the population 40%, prevalence in the subpopulation without CR 50%, prevalence in CR 26%, the heritability for liability 22.1% was as follows:


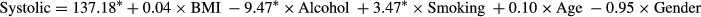
(7)

Note that adjusted *R*-squared for equation (7) was 0.19. In contrast, the regression model found from the sampling without CR (sample size 290) was as follows:


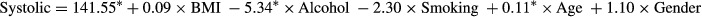
(8)

Note that adjusted *R*-squared for equation (8) was 0.16. Compared with equations (7) and (8), the results showed that the presence of CR is apparently affected by estimating regression coefficient in regression modeling (Table 4). By testing the assumption of parallel regressions for equations (7) and (8), the difference between equations (7) and (8) was significant (*F*-value = 24.96 *P*-value = 7.219e^−07^).

**Table 4:** Results of the regression coefficients between systolic blood pressure and environmental risk factors in the sample with and without cryptic relatedness (CR): sample size 500, ratio of CR to the population 42%, population prevalence 40%, heritability for liability 22.1%, prevalence in the subpopulation without CR 50%, prevalence in CR 26%

	Estimated	Standard error	*t*-value	Pr(>|*t*|)
Intercept				
Sample with CR^1^	137.18	4.91	27.92	<2e^−16^
Sample without CR^2^	141.55	2.99	47.26	<2e^−16^
BMI				
Sample with CR	0.04	0.14	0.29	0.77
Sample without CR	0.09	0.08	1.11	0.27
Alcohol consumption				
Sample with CR	−9.47	1.59	−5.94	5.47e^−09^
Sample without CR	−5.34	1.00	−5.35	4.26e^−07^
Smoking status				
Sample with CR	3.47	0.64	5.45	8.10e^−08^
Sample without CR	−2.30	0.44	−5.18	4.26e^−11^
Age				
Sample with CR	0.10	0.07	1.34	0.18
Sample without CR	0.11	0.05	2.39	0.018
Gender				
Sample with CR	−0.95	1.10	−0.94	0.35
Sample without vCR	−1.10	0.60	−1.83	0.07

1 Size of sample with CR was 500 subjects. From the sampling data with CR, equation (7) in Results were found. Adjusted *R*-squared = 0.19.

2 Size of sample without CR was 290 subjects. From the sampling data without CR, equation (8) in Results were found. Adjusted *R*-squared = 0.16.

The regression model found from the sampling data in the sample size of 500, the ratio of CR to the population 42%, prevalence in the population 50%, prevalence in the subpopulation without CR 67.2%, prevalence in CR 26%, the heritability for liability 31.7% was as follows:


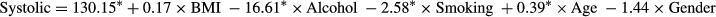
(9)

Note that size of sample with CR was 500 subjects and adjusted *R*-squared for equation (9) was 0.21. In contrast, the regression model found from the sampling without CR (sample size 290) was as follows:


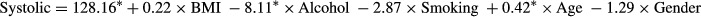
(10)

Note that adjusted *R*-squared for equation (10) was 0.24. Compared with equations (9) and (10), the results showed that the presence of CR is apparently affected by estimating regression coefficient in regression modeling (Table 5). By testing the assumption of parallel regressions for equations (9) and (10), the difference between equations (9) and (10) was significant (*F*-value = 35.46 *P*-value = 3.915e^−09^).

**Table 5:** Results of the regression coefficients between systolic blood pressure and environmental risk factors in the sample with and without cryptic relatedness (CR): sample size 500, ratio of CR to the population 42%, population prevalence 50%, heritability for liability 31.7%, prevalence in the subpopulation without CR 67.2%, prevalence in CR 26%

	Estimated	Standard error	*t*-value	Pr(>|*t*|)
Intercept				
Sample with CR^1^	130.15	5.51	23.62	<2e^−16^
Sample without CR^2^	128.16	3.92	32.73	<2e^−16^
BMI				
Sample with CR	0.17	0.15	1.13	0.26
Sample without CR	0.22	0.10	2.13	0.03
Alcohol consumption				
Sample with CR	−16.61	1.67	−9.95	<2e^−16^
Sample without CR	−8.11	1.24	−6.53	2.99e^−10^
Smoking status				
Sample with CR	2.58	0.71	3.63	0.0003
Sample without CR	−2.87	0.65	−4.40	1.53e^−05^
Age				
Sample with CR	0.39	0.08	4.91	1.21e^−06^
Sample without CR	0.42	0.05	7.72	1.99e^−13^
Gender				
Sample with CR	−1.44	1.09	−1.33	0.19
Sample without CR	−1.29	0.72	−1.79	0.08

1 Size of sample with CR was 500 subjects. From the sampling data with CR, equation (9) in Results were found. Adjusted *R*-squared = 0.21.

2 Size of sample without CR was 290 subjects. From the sampling data without CR, equation (10) in Results were found. Adjusted *R*-squared = 0.24.

## Discussion

We detected 20.2 % CR of the sample in Takahata cohort study. In our multiple regression models using sample size (*N* = 1000), there is no significant difference of regression coefficients in the sample with and without CR. In contrast, in the case that the population prevalence of SBP 40–50%, the prevalence in the subpopulation without CR 50–76%, the ratio of CR to the population 42–52%, sample size 400–500 and the heritability for liability 22–32%, the confounding effect of CR in the relationship between SBP and environmental risk factors is not negligible. In general, confounding is a major concern in causal studies because it results in biased estimation of exposure effects. In this respect, our study showed that confounding due to CR biased the estimation of exposure effects in the case of the heritability for liability by approximately >20%. On the other hand, although the number of predictors in the models were included enough (i.e., using independent variables for BMI, alcohol consumption, and smoking status, which were significantly correlated with blood pressure in several research groups (Kannel 1967; Minami et al. 1999; World Health Organization 2000; Ohira et al. 2009), adjusted *R*-square values of our regression equation models were not high. A possible explanation of low adjusted *R*-square values for our models is that other independent variables due to genetic factors might contribute to the SBP phenotype. Genetic factors that confer susceptibility to hypertension were identified in several populations (Jeunemaitre et al. 1992; Hata et al. 1994; Lifton 1996; Cusi et al. 1997). Theoretically, Fisher (1918) indicated that the impact of the effect on the phenotype was evaluated by comparing variances. Falconer and Mackay (1996) showed how the phenotypic variance can be partitioned into causal components of variance using the equation *V*_P_ = *V*_G_ + *V*_E_. In this sense, *V*_P_ is the total phenotypic variance, *V*_G_ is the total genetic variance consistent with the additive variance (*V*_A_), the dominance variance (*V*_D_), the interaction variance (*V*_I_) and *V*_E_ as the environmental variance consistent with the special environmental variance (*V*_Es_) that refers to the within individual variance arising from localized circumstances, and general environmental variance *V*_Eg_ refers to the environmental variance contributing to between-individual component in origin. Note that the ratio *V*_G_ = *V*_P_ is the heritability of the character. Moreover, Falconer and Mackay (1996) revealed the existence of two coefficients for genetic variances; one is the coefficient *r* of the additive variance (*V*_A_) which called the coefficient of relationship between the relatives in question, and the other is the coefficient *u* of the dominance variance (*V*_D_) which represents the probability of the relatives having the same genotype through IBD. Using these two coefficients, the total genetic variance is given by *V*_G_ = *rV*_A_ + *uV*_D_ + 2cov_AD_, where cov_AD_ is the covariance of breeding values with dominance deviations (Falconer and Mackay 1996). According to this mathematical model, it is easily understood how factors associated with genetically close relatives in a sample of ostensibly unrelated individuals contribute to the effect of phenotype; that is, the phenotype is composed of both environmental and genetic elements that contribute to the relationship between relatives. Thus, some of the differences in the estimates of regression coefficients might be because of the adjustment strategies for concomitant confounding effect of CR. Rotimi et al. (1999) examined a familial pattern of blood pressure in a population of Nigerian families and clarified that heritability of <50% for both SBP and diastolic blood pressure (DBP) reinforced the importance of the nonshared familial environmental effects. Thus, one of the approaches to select the best model for the response variable using collected cohort data from a limited or small-sized sample is that the heritability of the blood pressure phenotype might be worth considering.

Historically, the most common statistical approach for dealing with confounding in epidemiology was based on stratification. Typically, given the importance of confounding in epidemiology, statistical methods recommend the removal of significantly confounding samples. However, the resulting removal of samples with confounding factors, the sample size is reduced. As with another possible approach, we are now extensively analyzing this issue by a method incorporating principal components of a large subset of GWAS SNPs as regression covariates. This approach does not waste resources; that is we can use the entire sample. There are some similarities between the approach by Price et al. (2006) and our method; however, in contrast we examine principal components of a large subset of GWAS SNPs to adjust the confounding effect of CR. On the other hand, they examined them only to adjust the population structure. Generally, principal components typically reflect genome-wide factors attributable to the demographic history of the populations studied (Price et al. 2006). In this respect, it still remains to be clarified whether principal components reflect genome-wide factors attributable to CR. We are now extensively analyzing under what condition such approach is plausible.

In conclusion, we found a confounding effect of CR in the relationship between SBP and environmental risk factors was not negligible. In our study, we showed that heritability for liability might reflect on the estimation of regression coefficients between SBP and environmental risk factors, because they vary with environmental risk factors that differ across some unsuspected relatedness. For the genetic case-control studies, test statistics are generally inflated relative to the expectation under the association of an independent sample and without genetic association to the disease. These false positives often are attributed to CR (Devlin and Roeder 1999). Thus, more or less in any other epidemiological investigations that were performed previously, a true effect might be hidden due to confounding arising from CR. In our study, we presented a simply modeling to illustrate the effect of CR on the estimation of coefficients. The size of the CR would have a big impact on the precision of the resulting estimates of coefficients. We are now extensively analyzing this issue in different settings. Various statistical methods have been proposed to take into account confounding factors such as linear mixed-effect models (Demidenko 2004) or methods that adjust data based on a principal components analysis (Price et al. 2006). Sturmer et al. (2005) proposed a method of adjusting for multiple unmeasured confounders in a cohort study. The amount of residual confounding due to unmeasured and poorly measured covariates was important enough to qualitatively change the association between NSAID (nonsteroidal anti-inflammatory drug) use and mortality (Sturmer et al. 2005). After data collection, using these techniques in an epidemiological association study, it might be important to adjust the cryptic relative pairs based on genetic data in the relationship between environmental risk factors and phenotypes.

## Appendix

The following are the contributors of Yamagata University Genomic Cohort Consortium

**Steering Committee:** Takamasa Kayama^1^ (Chair), Hidetoshi Yamashita^2^ (Deputy chair), Akira Fukao^3,4^, Isao Kubota^5,6^, Takeo Kato^7,8^, Chifumi Kitanaka^9,10^, Shinya Sato^1,5^, Yoshiyuki Ueno^11^

**Respiratory, Cardiovascular, and Renal Diseases and Related Traits:** Isao Kubota^5,6^, Shinya Sato^1,5^, Tsuneo Konta^6^, Yoko Shibata^6^, Tetsu Watanabe^6^, Shuichi Abe^6^, Takuya Miyamoto^6^, Sumito Inoue^6^, Takehiko Miyashita^6^, Kazunobu Ichikawa^6^, Tetsuro Shishido^6^, Takanori Arimoto^6^, Hiroki Takahashi^6^, Satoshi Nishiyama^6^, and Ami Ikeda^6^

**Metabolic and Neurodegenerative Diseases and Related Traits:** Takeo Kato^7,8^, Makoto Daimon^8^, Toru Kawanami^8^, Manabu Wada^8^, Shigeki Arawaka^8^, Hidetoshi Oizumi^8^, Katsuro Kurokawa^8^, Shingi Susa^8^, Yuichi Katou^8^, Wataru Kameda^8^, Shingo Koyama^8^, Shigeru Karasawa^8^, Chifumi Iseki^8^, and Yoshimi Takahashi^8^

**Cancer and Related Traits:** Chifumi Kitanaka^9,10^, Yoshiyuki Ueno^11^

**Gastrointestinal, Hepatic, and Pancreatic Diseases and Related Traits:** Yoshiyuki Ueno^11^, Sumio Kawata^11^*, Takafumi Saito^11^, Naohiko Makino^11,12^, Kazuo Okumoto^11^, Hiroaki Haga^11^, Takeshi Sato^11^, Chikako Sato^11^, Hisayoshi Watanabe^11^, Yuko Nishise^11^, Rika Ishii^11^, Akiko Matsuda^12^, and Tomohiro Tozawa^11,12^

**Eye Diseases and Related Traits:** Hidetoshi Yamashita^2^ and Kei Honma^2^

**Cohort Establishment:** Akira Fukao^3,4^, Hiroto Narimatsu^3^, Kyoko Shibata^3^, Akiko Miura^3^, Rina Inoue^3^, Ai Numazawa^3^, Kahori Kudo^3^, Yoko Aita^3^, Noriko Umezawa^3^, Yuko Saito^3^, Yumi Takahashi^3^, Yuka Suzuki^3^, Katsumi Otani^4^, Atsushi Hozawa^4^, Li Shao^4^, Masatsugu Orui^13^, Atsuko Kobayashi^14^, Yuka Kanoya^14^, Takiko Hosoya^14^, Ikuko Suzuki^14^, Mariko Otake^14^, Yuko Morikagi^14^, Akiko Sekimata^14^, Manami Hiraka^14^, Yumi Matsuda^14^, Chika Sato^14^, Yoko Takeda^14^, Yoko Matsunami^14^, Tatsuya Horie^14^, Shiho Sato^14^, Mizue Inoue^14^, and Kaoru Baba^14^

**Genetic, Genomic, and Statistical Analyses:** Gen Tamiya^15^, Masao Ueki^15^, and Tomohiro Nakamura^15^

**Genotyping:** Gen Tamiya^15^, Jamiyansuren Jambaldorj^16^, and Satoko Araki^16^

**DNA Extraction and Biobanking:** Osamu Nakajima^17,18^

**Database Construction:** Kazuei Takahashi^19^ and Kazuo Goto^19^

**Patent Control and Commercialization:** Kimishige Ishizaka^20^

1. Yamagata University, Graduate School of Medicine, Department of Neurosurgery
2. Department of Ophthalmology
3. Advanced Molecular Epidemiology Research Institute (AMERI), Cohort Management Unit
4. Department of Public Health
5. AMERI, Respiratory and Cardiovascular Diseases Research Center
6. Department of Cardiology, Pulmonology, and Nephrology
7. AMERI, Metabolic and Degenerative Diseases Research Center
8. Department of Neurology, Hematology, Metabolism, Endocrinology and Diabetology
9. AMERI, Oncology Research Center
10. Department of Molecular Cancer Science
11. Department of Gastroenterology
12. Division of Endoscopy, Yamagata University Hospital
13. Yamagata Prefectural Tsuruoka Hospital
14. School of Nursing
15. AMERI, Genomic Information Analysis Unit
16. AMERI, Shared Laboratory
17. AMERI, Specimen Management Unit
18. Research Laboratory for Molecular Genetics
19. AMERI, Data Management Unit
20. COME center

*Present address: Hyogo Prefectural Nishinomiya Hospital.
